# Evaluation of Docking Machine Learning and Molecular Dynamics Methodologies for DNA-Ligand Systems

**DOI:** 10.3390/ph15020132

**Published:** 2022-01-22

**Authors:** Tiago Alves de Oliveira, Lucas Rolim Medaglia, Eduardo Habib Bechelane Maia, Letícia Cristina Assis, Paulo Batista de Carvalho, Alisson Marques da Silva, Alex Gutterres Taranto

**Affiliations:** 1 Department of Bioengineering, Federal University of Sao Joao del-Rei, Praça Dom Helvecio, 74, Fabricas, Sao Joao del-Rei 36301-1601, MG, Brazil; lucas.medaglia@hotmail.com (L.R.M.); leticiaassisquimica@hotmail.com (L.C.A.); 2 Federal Center for Technological Education of Minas Gerais, Department of Informatics, Management and Design, CEFET MG, Campus Divinopolis, Rua Alvares de Azevedo, 400, Bela Vista, Divinopolis 35503-822, MG, Brazil; habib@cefetmg.br (E.H.B.M.); alisson@cefetmg.br (A.M.d.S.); 3 Feik School of Pharmacy, University of the Incarnate Word, 4301 Broadway, San Antonio, TX 78209, USA; pcarvalh@uiwtx.edu; 4 Faculty of Computing, University of Latvia (UL), Raina Boulevard 19 Center District, LV-1050 Riga, Latvia

**Keywords:** computer drug design, molecular docking, molecular dynamic simulation, virtual screening, MolAr, DNA intercalating agents

## Abstract

DNA is a molecular target for the treatment of several diseases, including cancer, but there are few docking methodologies exploring the interactions between nucleic acids with DNA intercalating agents. Different docking methodologies, such as AutoDock Vina, DOCK 6, and Consensus, implemented into Molecular Architect (MolAr), were evaluated for their ability to analyze those interactions, considering visual inspection, redocking, and ROC curve. Ligands were refined by Parametric Method 7 (PM7), and ligands and decoys were docked into the minor DNA groove (PDB code: 1VZK). As a result, the area under the ROC curve (AUC-ROC) was 0.98, 0.88, and 0.99 for AutoDock Vina, DOCK 6, and Consensus methodologies, respectively. In addition, we proposed a machine learning model to determine the experimental ∆T_m_ value, which found a 0.84 R^2^ score. Finally, the selected ligands mono imidazole lexitropsin (**42**), netropsin (**45**), and *N*,*N*′-(1H-pyrrole-2,5-diyldi-4,1-phenylene)dibenzenecarboximidamide (**51**) were submitted to Molecular Dynamic Simulations (MD) through NAMD software to evaluate their equilibrium binding pose into the groove. In conclusion, the use of MolAr improves the docking results obtained with other methodologies, is a suitable methodology to use in the DNA system and was proven to be a valuable tool to estimate the ∆T_m_ experimental values of DNA intercalating agents.

## 1. Introduction

Drugs interacting with DNA are among the most effective anticancer agents [1], but their low selectivity makes them highly toxic, a major drawback that calls for new studies and strategies to develop drugs selective towards DNA in cancerous cells [2].

One of the strategies for the development of new drugs is to identify small molecules through a systematic analysis of large groups of compounds with drug-like properties. An experimental approach commonly used is the high throughput screening (HTS), an automated process using robots for a systematic search. It is a costly technique due to the number of compounds to be acquired, the cost of purchase and operation of sophisticated robots [3], and experimental considerations such as stability and solubility of the compounds.

An alternative to HTS is the virtual high-throughput screening (vHTS or VS), an in silico method to test large groups of compounds, including databases available online containing millions of molecules. This technique also allows the design and virtual testing of theoretical compounds prior to synthesis or acquisition, reducing the cost and time required to find compounds with a high potential for further development [3,4]. VS methods use molecular docking to study the interaction between small molecules and their receptors [5], a method that has been evaluated for protein-ligand systems, and more recently have been used to model DNA-ligand complexes [6,7,8]. However, most docking programs use algorithms that are not suitable for modeling DNA due to its high charge density [1], prompting the need for a more adequate in silico model for nucleic acids.

Several studies have been done trying to develop a molecular docking software appropriate for DNA modeling. Ricci and Netz [9] developed a method to predict the binding mode of small molecules to DNA using AutoDock 4.0 [10], which used distinct DNA receptors in the most common conformations related to the most common binding poses to suppress the importance of the receptor’s flexibility in the algorithm.

Srivastava et al. [11] described a systematic computational analysis of 57 DNA ligands through four docking protocols, with the following root-mean-square deviation (RMSD) for the best ligands: GOLD [12] (1.24 Å), Glide [13] (1.23 Å), CDOCKER [14] (1.44 Å), and AutoDock [10] (1.57 Å). GOLD and GLIDE, with similar values, were shown to have a better performance and being the most suitable for modeling nucleic acid-ligand complexes. Molecular dynamics simulations showed that the DNA duplex skeleton underwent minor deviations in the complex, supporting docking protocols even though the receptor is kept rigid. However, the area under the ROC curve (AUC) of these methodologies was not evaluated. ROC curve is an important metric to check the capacity of methodology to distinguish false positive results. Fong and Wong [15] evaluated four scoring functions (AutoDock [10], ASP@GOLD [16], ChemScore@GOLD [17], and GoldScore@GOLD [12]) for DNA-ligand complexes, and the scoring functions reproduced the experimental crystallographic structure complexes. It is noteworthy that these previous studies improved their results by combining more than one scoring function.

Good RMSD results were obtained in previous studies, but the ranking capacity of these docking methods was not evaluated. Our study used Molecular Architecture (MolAr) [18] software to predict DNA-ligand poses. MolAr is a docking workflow that allows an integrated and automated virtual screening (VS) process, from protein preparation (homology modeling and protonation state) to virtual screening with different methods. MolAr is open access and free of charge (available at http://www.drugdiscovery.com.br, accessed on 20 May 2020), allowing users to perform all the docking steps in a unique interface with a simple and intuitive operation. It uses AutoDock Vina [19], DOCK 6 [20], and Consensus Virtual Screening (CVS) docking protocols. AutoDock Vina uses a hybrid scoring function that combines knowledge-based and empiric scoring function features. DOCK 6 offers physics-based energy score functions based on force fields and score functions (GRID and AMBER scores). CVS is a ranking normalized combination of the results of AutoDock Vina and DOCK 6, reducing the chance of false positive results [18]. Our results were evaluated for DNA-ligand model systems [11] through visual inspection of the RMSD and ROC curves. In addition, docking binding energy and descriptor values of ligands were used as a predictor to calculate the ∆T_m_ experimental values of 11 DNA ligands previously reported. Dynamic molecular simulations were also used to clarify their intermolecular interactions with DNA.

## 2. Results

**Figure 1:**
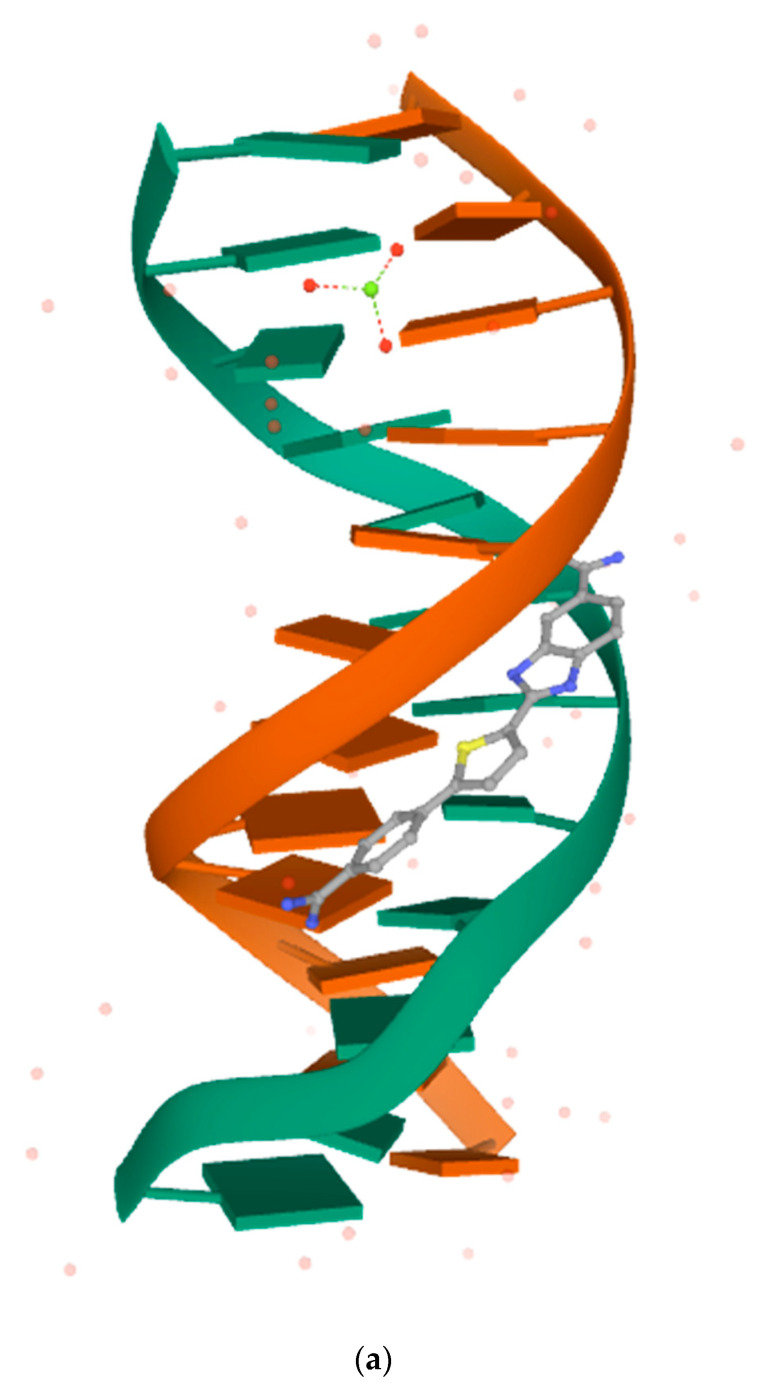

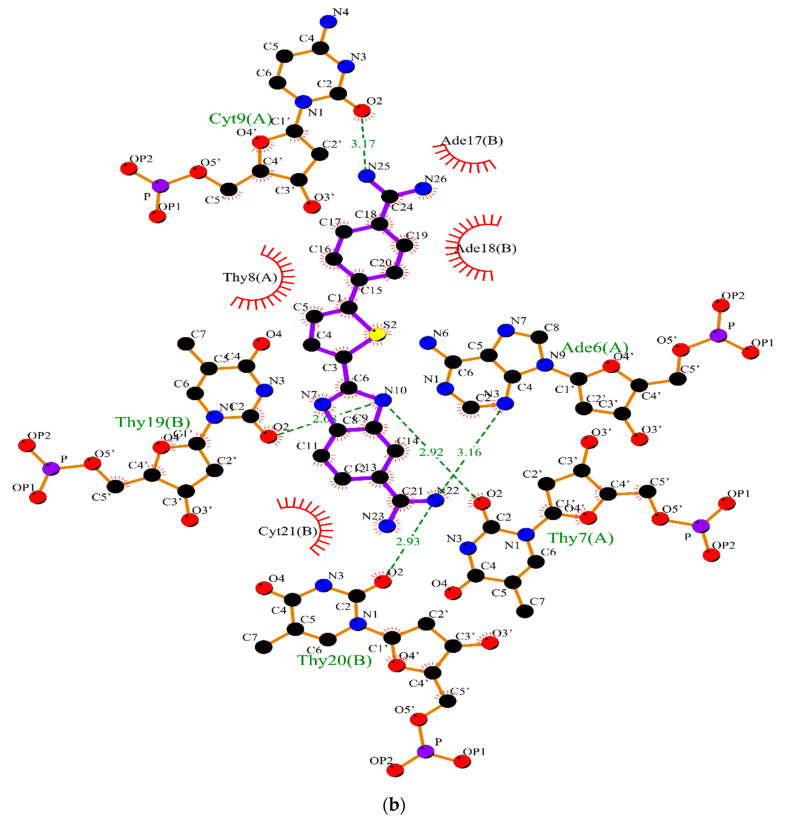
(**a**) The crystallographic structure of molecular target under PDB code 1VZK; (**b**) a close view of the intermolecular interactions between ligand (D1B) in the minor DNA groove of the 1VZK. The red circles and ellipses in each plot indicate protein residues. Hydrogen bonds are shown as green dotted lines, while the spoked arcs represent residues making van der Waals interactions with the ligand generated with LigPlot+.

A visual inspection was performed on the 1VZK structure to identify the principal forces for molecular recognition. Figure 1 shows the 3D structure of the target 1VZK and a 2D diagram with its crystallographic ligand (D1B). The ligand is complexed into the minor groove of DNA (Figure 1a) through hydrogen bonds between amidinic moieties and the carbonyl oxygen of nitrogenous bases. Hydrophobic interactions can also be observed between the benzimidazole and aromatic and with nitrogenous bases (Figure 1b).

In general, redocking is the first evaluation method to be used in the docking process. This process shows (i) the correct elaboration of grid box parameters; (ii) the capacity of the docking method of reproducing the crystallographic binding pose; (iii) the acquisition of binding energies that can be used to rank the compounds. Usually, the redocking is evaluated by the RMSD value between the crystallographic binding pose and redocking results. The RMSD value between 1VZK and D1B ligand was 0.65 Å by AutoDock Vina, while the threshold value is 2.0 Å [20]. This result was better than previous docking methodologies, GOLD, GLIDE, CDOCKER, and AUTODOCK, which had values ranging from 1.23 Å to 1.57 Å [11].

### 2.1. Molecular Docking 

The docking performance was evaluated by calculating the AUC-ROC, EF values, and BedROC. AUC-ROC has been used to check if the docking method can distinguish false positives from true positives [21]. The AUC-ROC values for our test compounds were 0.98, 0.88, and 0.99 for AutoDock Vina, DOCK 6 (Amber Score), and CVS, respectively. Moreover, the enrichment factor (EF) value [22] reflects the ability of the docking calculations to find true positives throughout the background database compared to random selection. Thus, it indicates how good the set formed by the top x% ranked compounds is compared to a set of equal size selected randomly from the entire collection of compounds. EFs values are calculated utilizing a percentage of the data set. For example, EF5% represents the value obtained when 5% of the database was screened. The EF value is defined by:

(1) $$\mathrm{EF}\;\%=\frac{\mathrm{actives}\;\%}{\mathrm{compounds}\;\%}\times \frac{\mathrm{total}\;\mathrm{compounds}}{\mathrm{total}\;\mathrm{actives}}$$

Previous reports show CVS having the best EF values, compared to DOCK 6 and AutoDock Vina, which can be explained by the use of AutoDock Vina output as the input for DOCK 6. Consequently, AUC-ROC had values corroborated by the EF values; in other words, the EF validates AUC-ROC results, especially with the performance at EF 1%, showing the CVS method could distinguish 100% of molecules [18].

The BedROC [23] value was calculated to confirm these AUC-ROC and EF results. BedROC uses exponential weighting to give early rankings more weight than the latest rankings of active compounds. The BedROC values were 0.60, 0.52, and 0.83 for AutoDock Vina, DOCK 6 (Amber Score), and CVS, respectively. As in AUC-ROC and EF the values of CVS are better than AutoDock Vina and DOCK6 (Amber Score).

Finally, Machine Learning was used to develop a model to predict ∆Tm experimental values. ΔT_m_ represents the change in the melting temperature of DNA upon drug binding, being directly correlated with the binding energy, and is a valuable tool to evaluate the docking results. Six algorithms of linear regression were implemented as follows: (i) Gradient Boosting Regressor [24]; (ii) Random Forest Regressor [25]; (iii) Linear Regressor [26]; (iv) Voting Regressor [27] between algorithms (i), (ii) and (iii), (v) Lasso [28] and (vi) Elastic Net [29]. The results are summarized in Table 1, with Mean Squared Error (MSE) and R^2^ score information. The Gradient Boosting Regressor shows the best result, with an R^2^ score of 0.84, and the worst is the Random Forest Regressor with an R^2^ score of 0.33.

### 2.2. Molecular Dynamics

**Figure 2:**
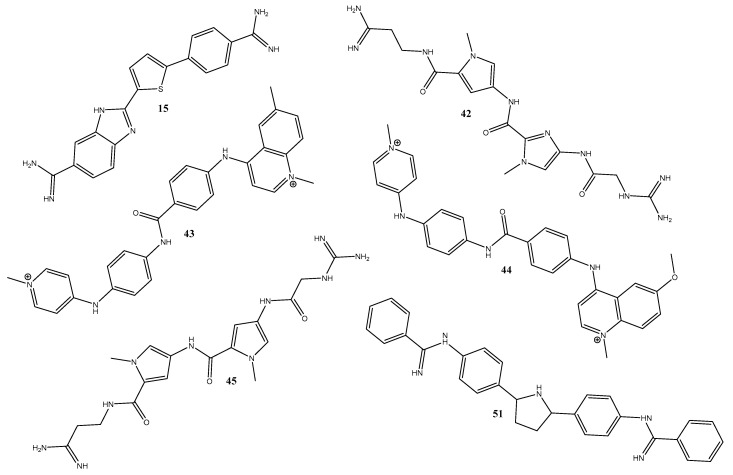
Ligands chosen for MD simulations after docking simulation.

The five best CVS results and the original ligand were chosen for simulation and presented in Figure 2, where the conformation changes of each of these ligands during the MD simulation were analyzed.

**Figure 3:**
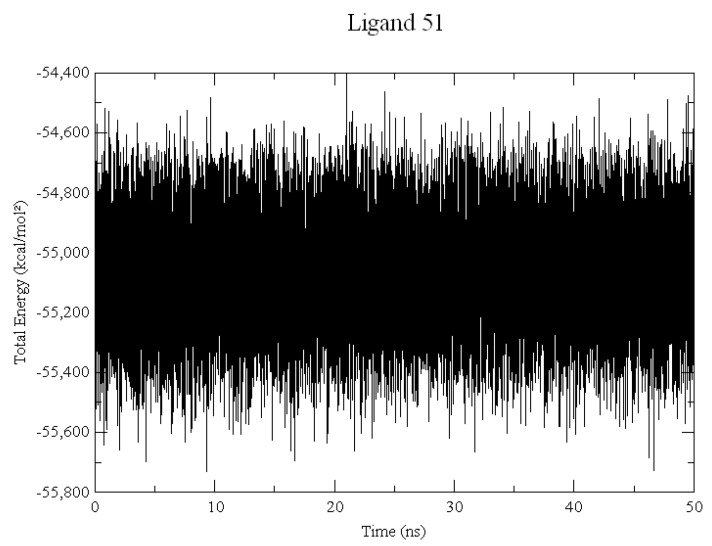
Example of Total Energy (Kcal/mol) calculated in Dynamic Molecular Simulation for interactions with DNA and ligand **51**.

The average energy for the total system, in Kcal/mol was −55,067.1, −55,473.3, −55,507.2, −55,137.1, −54,524.8, and −54,988.1 to ligands **51**, **42**, **45**, **15**, **43**, and **44**, respectively. The total energy graph shown in Figure 3 demonstrates an example of how the energy has a minimal variation. All energy graphs (available in the Supplementary Material, Figure S3) remained in equilibrium throughout the entire MD simulation.

**Figure 4:**
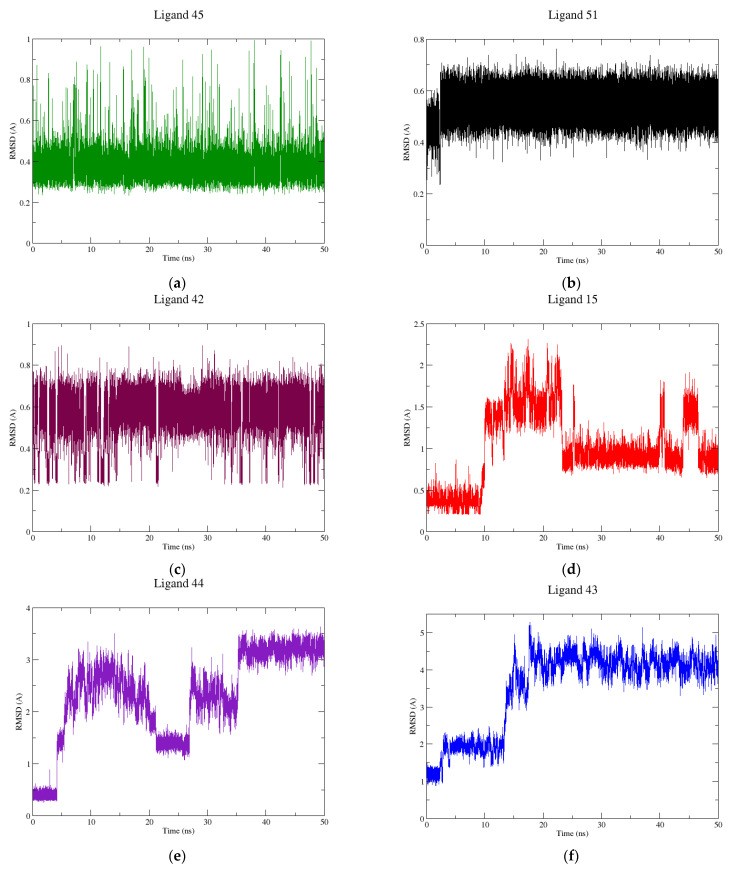
RMSD graphs for: (**a**) **45**; (**b**) **51**; (**c**) **42**; (**d**) **15**; (**e**) **44**; (**f**) **43** ligands complexed with DNA during 50 ns.

The simulations were carried out in 50 ns to observe if there were significant conformational changes during the trajectory, with the results summarized in Figure 4. As can be observed, the ligands 42, 45, and 51 have the best results with an RMSD variation below 1Å. The original ligand and ligands **43** and **44** showed a major variation of approximately 2, 5, and 4 Å, respectively.

**Figure 5:**
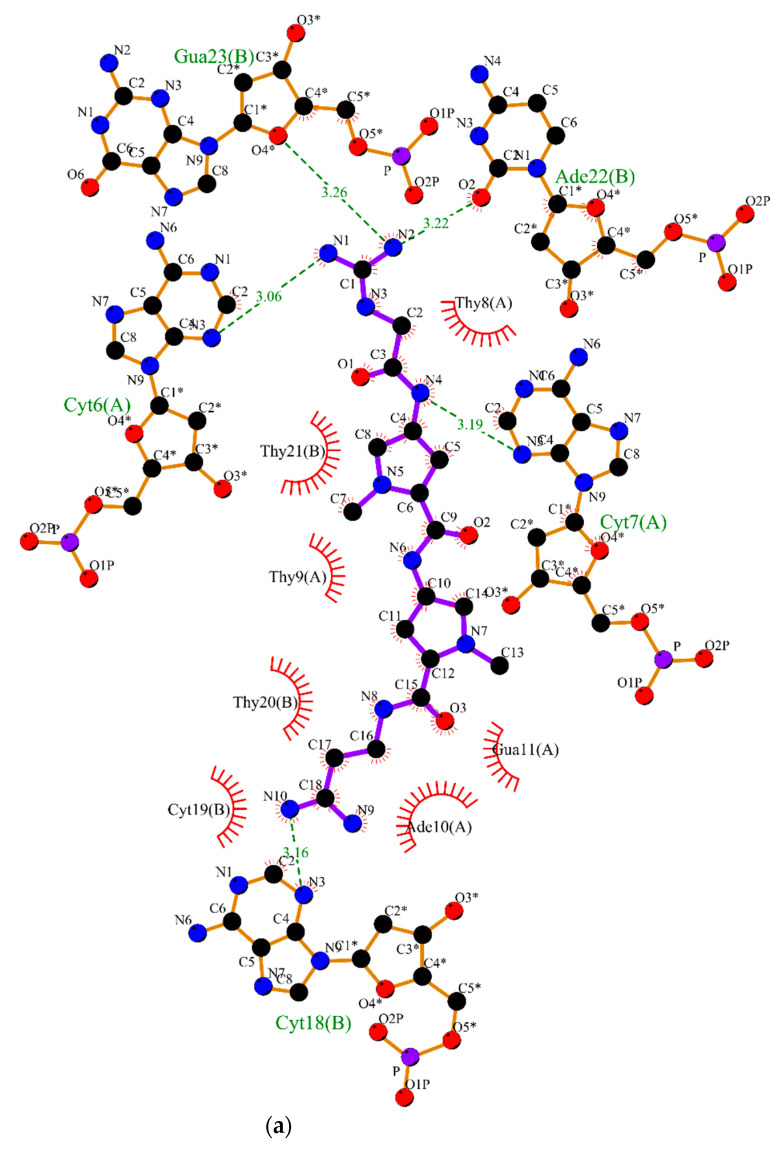

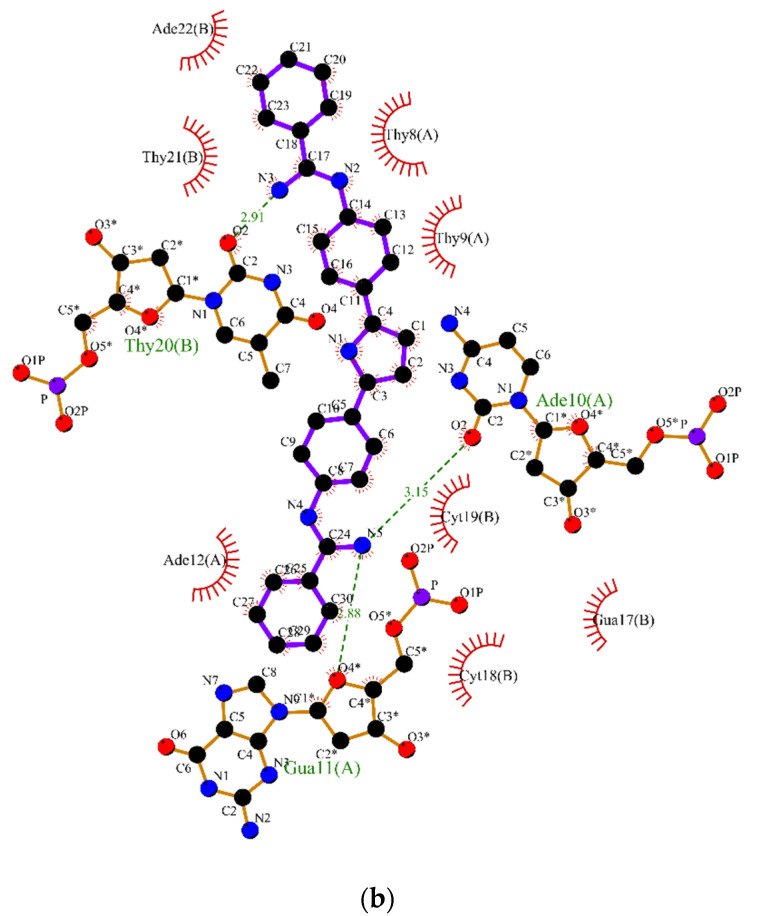

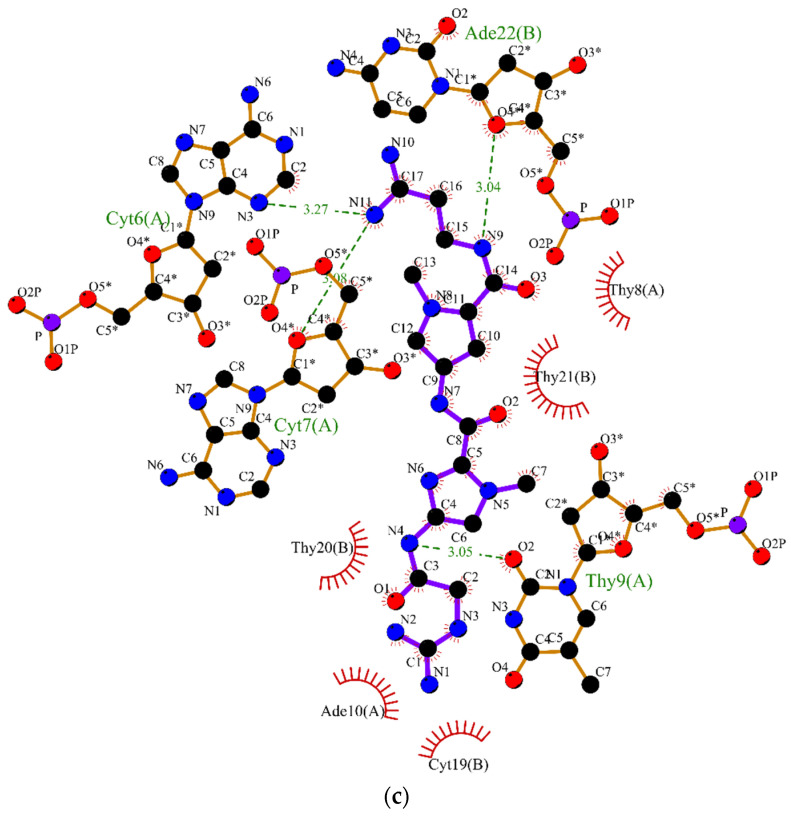

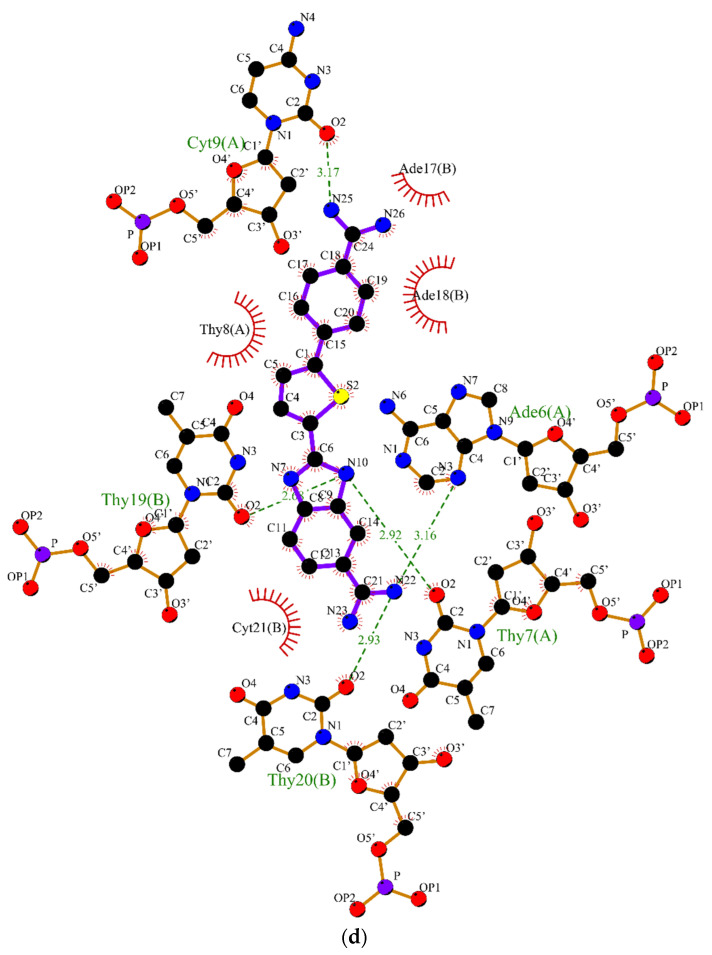

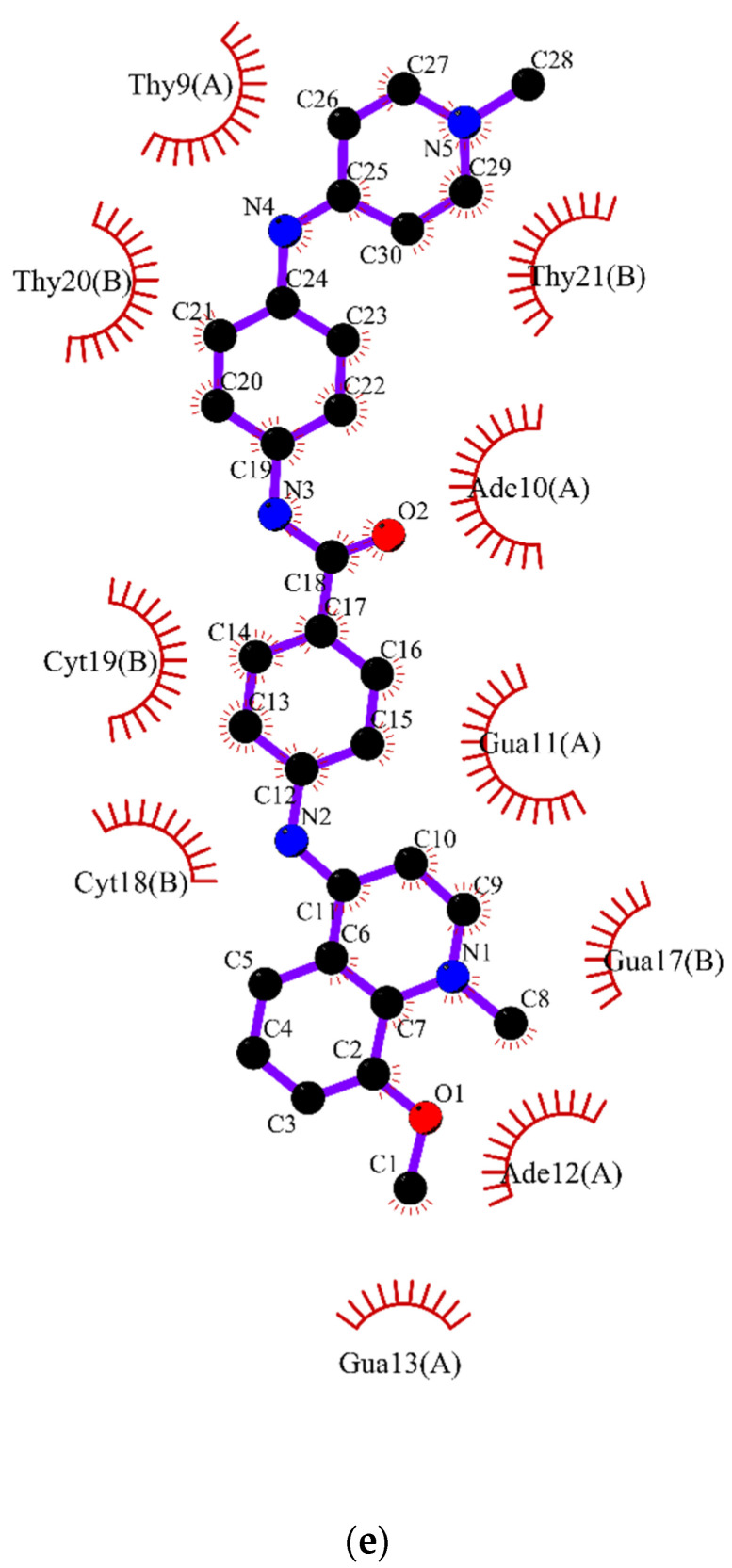

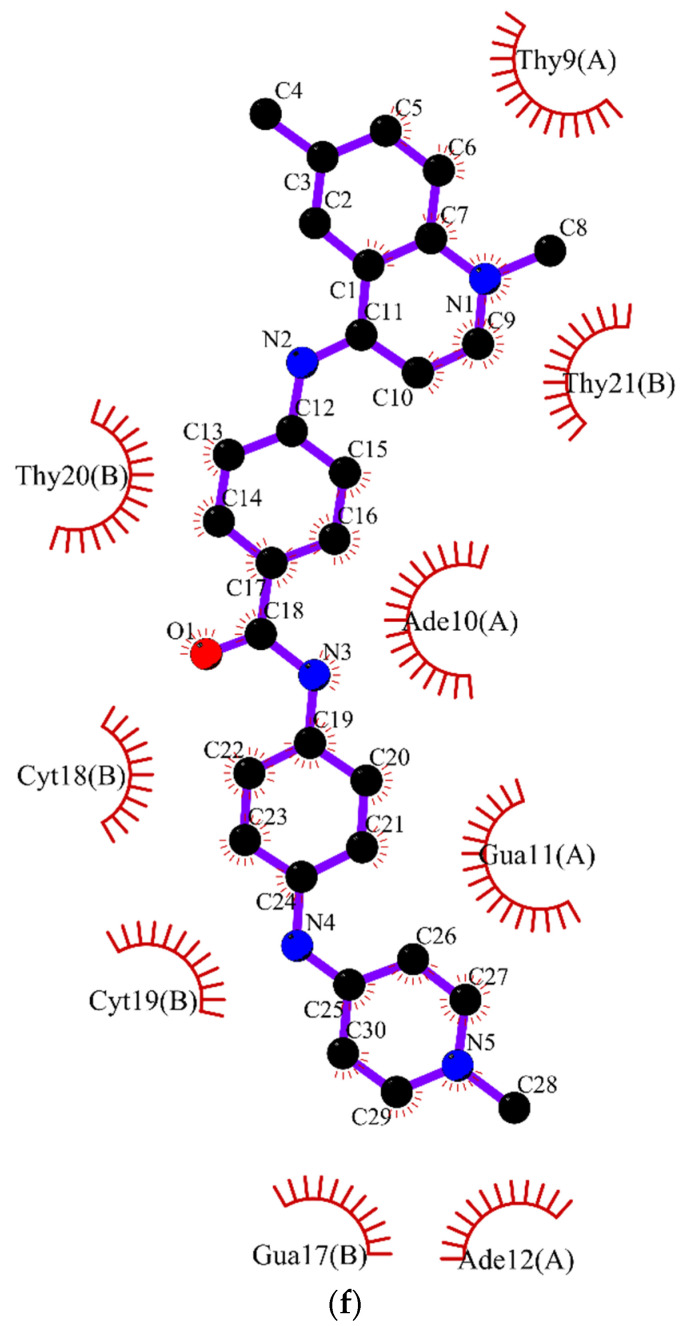
2D interaction diagram obtained by LigPlot+ in Dynamic Molecular Simulation for interactions with DNA and ligand: (**a**) **45**; (**b**) **51**; (**c**) **42**; (**d**) **15**; (**e**) **44**; (**f**) **43**. The red circles and ellipses in each plot indicate protein residues. Hydrogen bonds are shown as green dotted lines, while the spoked arcs represent residues making van der Waals interactions with the ligand.

The intermolecular interactions of structures in equilibrium can be observed in Figure 5. As can be observed, the ligands **45** (5a), **51** (5b), **42** (5c), **15** (5d) were able to form hydrogen bonds and van der Waals interactions; whereas the ligands **44** (5e) and **43** (5f) carried out van der Waals interactions with nucleic acids.

**Figure 6:**
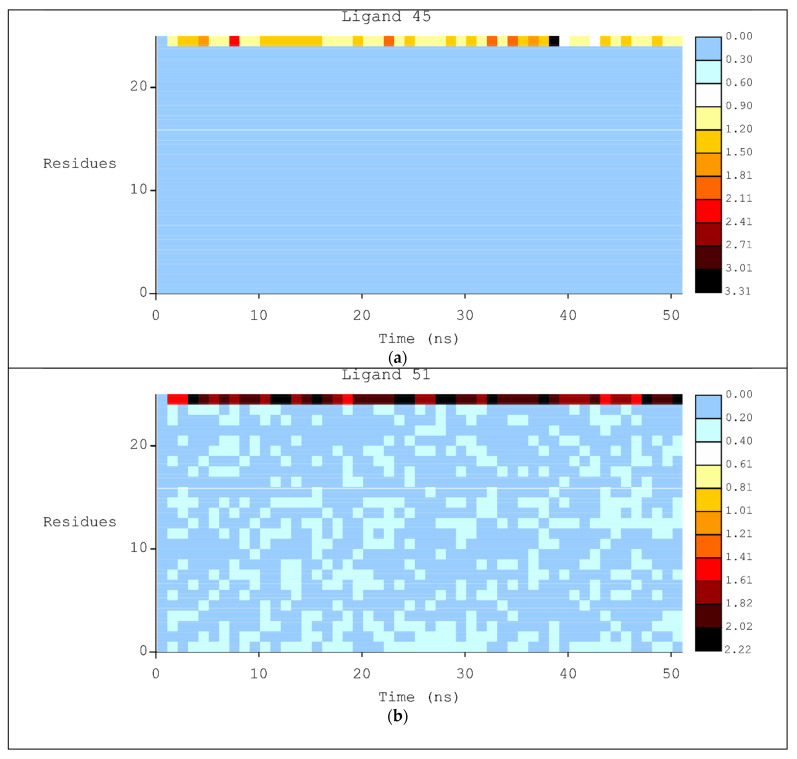

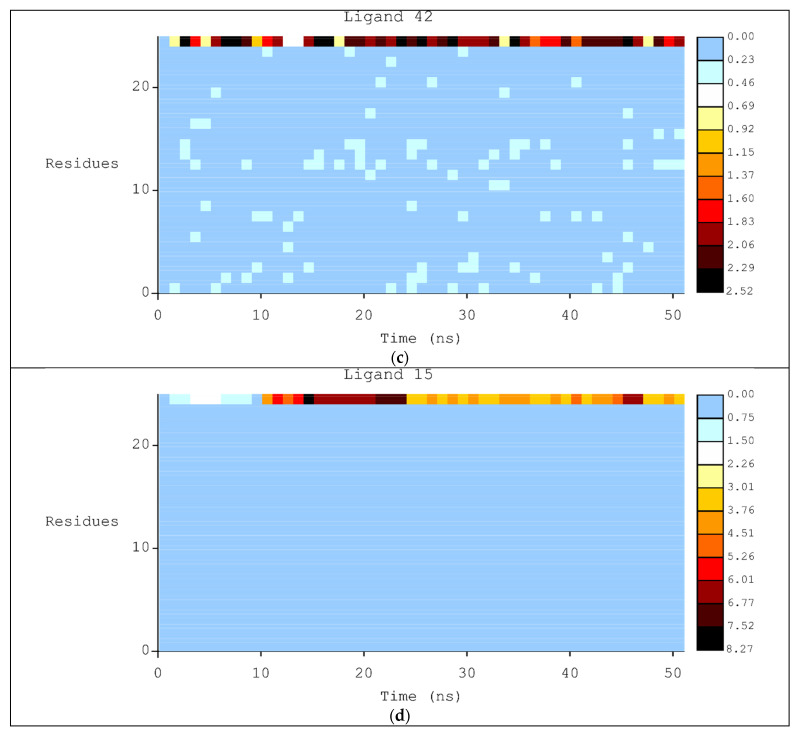
Heat Map for MD simulations generated with HeatMap plugin in VMD with ligand: (**a**) **45**; (**b**) **51**; (**c**) **42**; (**d**) **15**.

Finally, to improve the analysis of RMSD values fluctuation, the heat map was plotted with the best ligands using VMD software. Figure 6 shows the heat map for ligands **45**, **51**, **45**, and **15**, Figure 6a–d, respectively. In general, the DNA structure is kept rigid during the MD trajectory with low variation. However, the highest fluctuation can be observed for all ligands reaching values ranging from 1.2 to 8.27.

## 3. Discussion

### 3.1. The Best Docking Methodologies to Study the DNA System

In this study, we use AUC-ROC, EF, and BedROC to evaluate the best docking methodology for DNA intercalating agents, comparing AutoDock Vina, DOCK6, and CVS. AutoDock Vina is an important tool to find the correct pose of ligand into the binding site [19]; however, the ranking among the ligands has not been carried out properly. In addition, the score function of AutoDock Vina does not consider the charges. On the other hand, the score function of DOCK 6 [20], Amber score, includes the AM1-BCC charges of the system. Consequently, the AM1-BCC charges have been determined for a start pose obtained from AutoDock Vina output, improving the accuracy of charge calculations.

AUC-ROC was used to check if the docking method can distinguish false positives from true positives. AUC values close to 1 suggest good discrimination between false and true positives, whereas values closer to 0.5 show a random process, and values higher than 0.7 represent a good distinguishing power [21].

EF indicates how good the set formed by the top x% ranked compounds is when compared to a set of equal size selected randomly from the entire collection of compounds. EF corroborates AUC-ROC [18], yielding even better results with CVS methodology. BedROC calculated values were 0.60, 0.52, and 0.83 for AutoDock Vina, DOCK 6 (Amber Score), and CVS, respectively, confirming that CVS is the best methodology.

The model of linear regression summarized in Table 1 shows the results of the implementation of six linear regressors. The Gradient Boosting Regressor shows the best result, with an R^2^ score of 0.84, and the worst is the Random Forest Regressor with an R^2^ score of 0.33. Gradient Boosting Regressor (GBR) is a generalization of boosting to arbitrary differentiable loss functions. GBR is an accurate and effective off-the-shelf procedure that can be used for both regression and classification problems in a variety of areas. Our GBR’s result is better than Srivastava’s studies [11], which used docking information from GOLD, GLIDE, CDOCKER, AUTODOCK 4, Average Information Content level 2, and chemical hardness. Thus, our Machine Learning model was able to estimate ∆T_m_ value with more accuracy than previous reports.

### 3.2. Molecular Dynamics Simulations

MD simulations were performed to obtain information about the ligands’ interaction and stability into the DNA groove. According to the results of total energy, ligand **51** obtained the lower energy; whereas ligand **44** obtained the highest energy value. Figure 3 demonstrates the lower variation of the system in simulation.

Figure 4 shows the RMSD results of MS simulations. Ligands **15**, **42**, **45,** and **51** showed a RMSD average variation below 1 Å. Both ligands **42** and **45** achieved the equilibrium state in the beginning of the process (Figure 4a,c). Noteworthy that ligand **51** (Figure 4b) showed a RMSD average value of 0.5 Å, indicating an absence of conformational changes for this inhibitor, suggesting a better molecular recognition between DNA and ligand in the DNA groove. In addition, **15** showed an RMSD average variation of 1 Å and stabilized in the DNA groove after 20 ns of simulation, as shown in Figure 4d. Visual inspection of the MD simulation path of **15** showed a decrease in the intermolecular interaction forces until 20 ns, followed by complete filling of the binding site during the rest of the process. **43** and **44** showed RMSD values higher than 2 Å, and it was observed that the 2-thienyl-1H-benzimidazole portion of **43** was more exposed to the solvent, resulting in a higher degree of freedom and consequent adoption of various conformations. **43** presented an RMSD value of 5.0 Å (Figure 4f), but reached equilibrium after 20 ns. **44** behaved similarly to **43**, reaching equilibrium at 35 ns (Figure 4e). **44** had the quinolinium group outside the major DNA groove, obtaining an RMSD value of 3.5 Å.

Summarizing, all ligands achieved equilibrium within 50 ns of simulation, characterizing molecular recognition. Although compounds ligand 43 and ligand 44 presented good docking results at 15 and 28 nanoseconds of dynamic simulation, respectively, the structures presented conformational changes, resulting from parts of the ligands leaving the DNA groove suggesting hydrogen bonding with the solvent.

MD results corroborate the molecular docking results, with compounds **42**, **45**, and **51** interacting and accommodating themselves better in the smaller groove of DNA, presenting themselves as promising compounds for further studies as anticancer drugs. Compounds **43** and **44** can be considered weak DNA intercalators because, despite good docking results, they had a higher variation of RMSD values during MD.

The molecular interactions are depicted on Figure 5. **45**, **51**, **42,** and **15** have hydrogen bonding acceptors and donors and were recognized by DNA through hydrogen bonds and van der Waals interactions (Figure 5a–d). For instance, Figure 4a shows the intermolecular interactions between compound **45** with DNA. This compound carried out hydrogen bonding with CytA:7, ThyA:9, AdeB:22 and hydrophobic interactions with ThyA:8, AdeA:10, and ThyB:20. Compound **51** (Figure 5b) was better recognized by the DNA minor groove by performing a higher number of intermolecular interactions, such as hydrogen bonding with AdeA:10, GuaA:11, and ThyB:20; and van der Waals interactions with ThyA:8, ThyA:9, AdeA:12, GuaB:17, CytB:19, and AdeB:22. Compound **42** (Figure 5c) is able to perform hydrogen bonding with CytA:6, CytA:7, ThyA:9, and AdeB:22; and hydrophobic interactions with ThyA:8, AdeA:10, CytB:19, and ThyB:20. It is noteworthy that the guanidinium groups of the **15** performed hydrogen bonds with CytA:6, CytA:7, CytB:18, AdeB:22, GuaB:23, beyond several hydrophobic interactions, such as GuaA:11, ThyB:20, and ThyB:21 (Figure 5d). These interactions with these nitrogenous bases are essential components for intercalation within the minor DNA groove, which indicates that this inhibitor remained well accommodated in the DNA during the dynamic’s simulation.

In contrast, both compounds **44** and **43** were not able to perform hydrogen bonding with DNA. **44** (Figure 5e) carried out hydrophobic interactions, for instance, with ThyA:9, AdeA:10, GuaA:11, AdeA:12, GuaA:13, GuaB:17, CytB:18, CytB:19, ThyB:20 and ThyB:21. Similarly, **43** performed hydrophobic interactions with ThyA:9, AdeA:10, GuaA:11, AdeA:12, GuaB:17, CytB:18, CytB:19 and ThyB:20, as shown in Figure 4f. These missing hydrogen bonding interactions can explain the higher RMSD fluctuations value during MD simulations, once this interaction has an important hole in the molecular recognition and stabilization of the ligand within the DNA groove. These findings highlight the structure-activity relationship of guanidinium groups in the development of antineoplastic compounds.

Through the analysis of RMSD plots using heat map graphs (Figure 6) it is possible to confirm the results generated by RMSD and interactions maps. **51** presented the smallest RMSD variation (max of 2.22 Å), best RMSD graphics and total energy. **42**, **45**, and **15** demonstrated RMSD variation of 2.52 Å, 3.31 Å, and 8.27 Å, respectively.

## 4. Materials and Methods

### 4.1. Molecular Docking

The three-dimensional structures of 57 ligands were constructed using Marvin Sketch [30] and 150 decoys were generated by the DUD-E platform [31]. The DNA molecular target was obtained from the Protein Data Bank (PDB-1VZK). These ligands and targets were described in a previous report [11] and are available in the Supplementary Material, Figure S1 [31]. The ligands were refined by Run_Mopac [32] software using Parametric Method 7 (PM7) [33] and Eigenvector Following routine [34]. 

The target was prepared by Chimera [35] by:

- removing water molecules and magnesium ions;
- adjusting the protonation state at pH 7.4;
- assigning charges using AMBERff14SB and AM1-BBC;
- minimizing the structure using 100 steps for steepest descent and 10 steps for conjugate gradient, each step measuring 0.02 Å.

Finally, all compounds were docked against the 1VZK molecular target at the minor groove position using a grid box with 20 × 20 × 26 Å and atomic coordinates centered to 14.44 Å, 20.57 Å, and 8.64 Å, for x, y, and z, respectively. In order to evaluate the best docking methods for DNA docking, three virtual screening simulations using MolAr were performed through AutoDock Vina, DOCK 6 (Amber Score), and CSV. All methodologies were double-checked by redocking, measurement of the area under the Receiver Characteristic Operator (ROC) curve (AUC-ROC), Enrichment Factor (EF), and Boltzmann-Enhanced Discrimination (BedROC). The redocking process consisted of removing the crystallographic ligand, with subsequent docking of the ligands into the same binding site.

In addition, we developed a machine learning model with the docking binding energy results and molecular descriptors of the molecules hereby tested to calculate the ∆T_m_ experimental values. The data frame was elaborated using the 57 compounds described previously, from which only 11 had their ∆T_m_ values calculated [11]. The descriptors were obtained using the Mordred library [36], a molecular descriptor calculator. Afterward, six Linear Regression algorithms were performed with the following descriptors: Molecular Weight, cLogP, cLogS, Total Surface Area, Relative Polar Surface Area, Polar Surface Area, AutoDock Vina with major groove, AutoDock Vina with minor groove, $E_{homo}$, $E_{homo}^{-1}$, $E_{lumo}$, $E_{homo}^{+1}$, DOCK 6 with Amber Score, Consensus with Grid and Amber Score, Structural Information Content level 1, Bond Information Content level 1, and chemical hardness [$\eta =\left(E_{lumo}-E_{homo}\right)/2)$].

### 4.2. Molecular Dynamics

The five best-docked ligands (according to MolAr Consensus with Amber score) were chosen for MD simulations among the crystallographic reference ligands (PDB-ID 1VZK) to characterize the molecular recognition between ligands and DNA. The ligands (PDB-IDs 1VZK, 1LEY, 1ZPH, 1ZPI, 261D, and 2GYX) are shown in the Supplementary Material, Figure S2. All ligands and the energy values for all configurations are presented in the Supplementary Material, respectively, in Figure S1 and Table S1.

The ligand-DNA complexes were inserted into a 74.15 × 52.33 × 55.58 Å simulation box and solvated with TIP3P model water molecules [37]. Sodium chloride ions were added to neutralize the system charge. Each system was energetically minimized with 5000 cycles using the Conjugate Gradient algorithm [38]. The nucleic acid atoms had position restraints with an exponent of energy function of 2 and scaling of 1.0 applied to them during the first 4000 cycles and no restraints during the last 1000 cycles. After the energy minimization, the systems were heated to 310 K during a 30 ps equilibration conducted under an isothermal-isochoric ensemble (NVT), followed by a 500 ps simulation under an isothermal-isobaric ensemble (NPT) using the Langevin piston method [39] to maintain the total pressure to an average of 1 bar. The final production had a total of 50 ns. Water stretching and bending motions were constrained by the SETTLE algorithm [40]. Electrostatic interactions were treated via the Particle-Mesh Ewald method [41,42] with a 12 Å cutoff radius. All simulations were performed using the CHARMM36 [41,43,44] force field implemented into NAMD software [33], version 2.13. Analysis was performed using VMD, version 1.9.3 [45].

## 5. Conclusions

Even though DOCK 6 and AutoDock Vina showed different results, the overall result was improved when they were combined and subjected to the MolAr CVS approach. AUC-ROC, BedROC, and EF values showed the combination was able to generate more reliable results and a better prediction of the ligand conformation. MD is a critical methodology to confirm the interactions between ligands and nucleic acids, showing that MolAr CVS virtual screening can rank ligands in the DNA intercalating compounds. It is noteworthy that CVS has a low computational cost when compared with MD simulations. 

In this study, two different approaches were carried out to predict the activity of compounds capable of binding to the minor groove of DNA. The first approach, structure-based drug design, was carried out to rank compounds for their ability to dock with the 1VZK molecular target at the minor groove position using docking and MD simulations. The second approach, ligand-based drug design through Machine Learning methods, ranked the six selected structures based on their binding energy. These methods were able to properly describe the intermolecular interactions between intercalating agents and DNA and build a machine learning model able to predict the ∆T_m_ experimental values. The application of docking machine learning and molecular dynamics methodologies suggests compounds **51**, **42**, and **45** as leads for the development of improved anticancer compounds.

## Figures and Tables

**Table 1:** Regression Linear values calculated for the Prediction of ∆T_m_ values.

Algorithm	MSE	R^2^ Score
Gradient Boosting Regressor	3.06	0.84
Random Forest Regressor	13.05	0.33
Linear Regressor	6.18	0.68
Voting Regressor	4.48	0.77
Lasso	7.88	0.59
Elastic Net	7.18	0.63

## Data Availability

Date is contained in the article and supplementary materials.

## Supplementary Materials

The following supporting information can be downloaded at: https://www.mdpi.com/article/10.3390/ph15020132/s1, Figure S1: Structure of all ligands with their PDB code and docking results values, Table S1: Docking Results with all configurations with energy values in Kcal/mol, Figure S2: Ligands chosen for MD simulations after docking. Figure S3. Total Energy (Kcal/mol) calculated in Dynamic Molecular Simulation for interactions with DNA and ligands: (a) **45**; (b) **51**; (c) **42**; (d) **15**; (e) **44**; (f) **43**.
