# Supplementary material for: Evaluation of Docking Machine Learning and Molecular Dynamics Methodologies for DNA-Ligand Systems
Source: Pharmaceuticals (Basel). 2022 Jan 22;15(2):132. doi: 10.3390/ph15020132 (PMC8874395; doi:10.3390/ph15020132)
Supplement: Supplementary file 1 [file pharmaceuticals-15-00132-s001.zip › pharmaceuticals-1508876-supplementary.pdf]

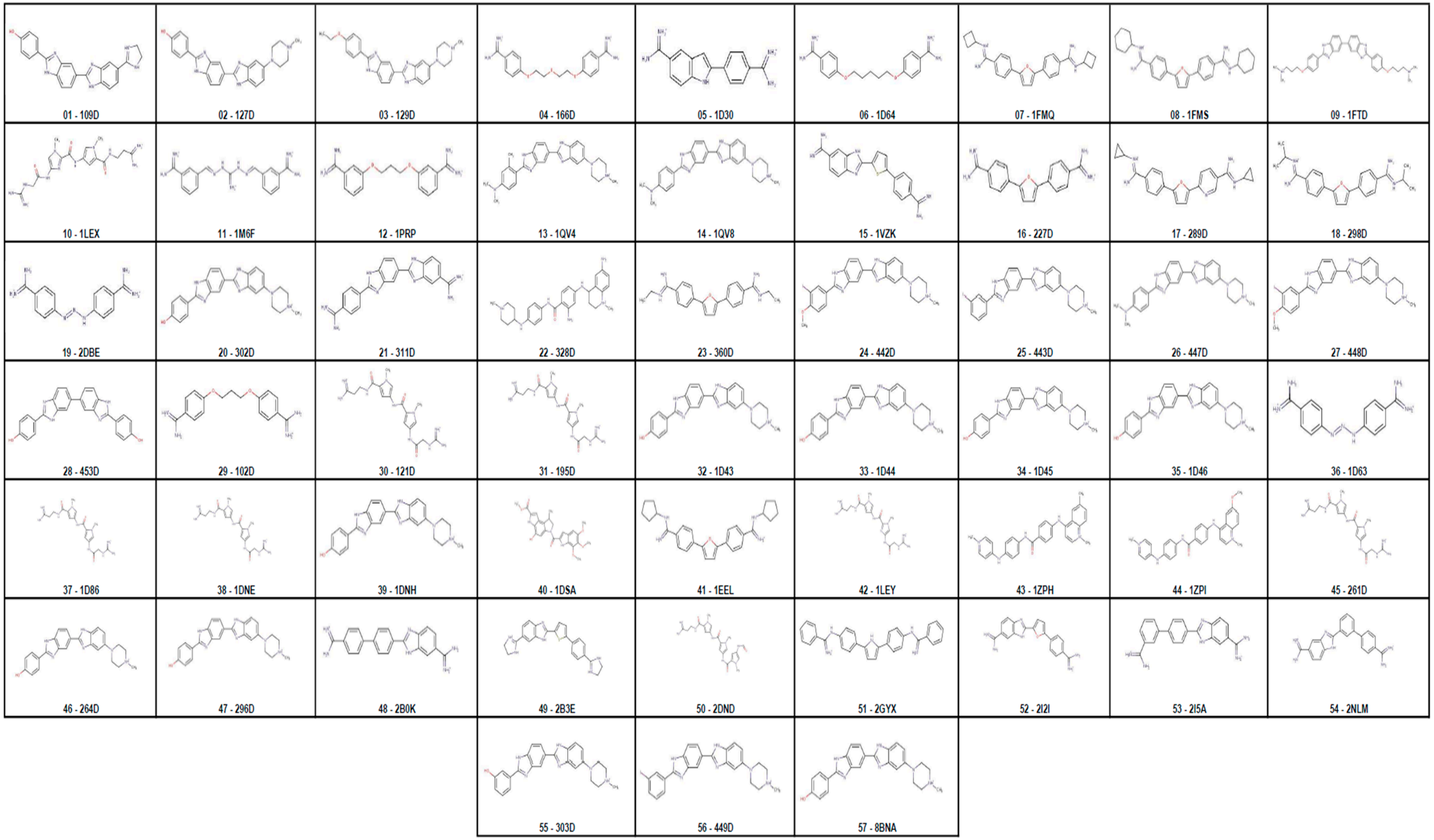

Figure S1. Structure of all ligands with their PDB code and docking results values.

Table S1. Docking Results with all configurations with energy values in Kcal/mol .

Ligand	Vina	D6A	CVS	Position
1 - 109D	-11.9	0[1]	10[1]	56th
2 - 127D	-11.8	-36.37	3.42	20th
3 - 129D	-11.5	-38.78	3.86	31st
4 - 166D	-7.9	-50.16	6.16	49th
5 - 1D30	-9.3	-50.43	4.94	45th
6 - 1D64	-8	-51.92	6.52	52nd
7 - 1FMQ	-9.6	-49.79	4.09	38th
8 - 1FMS	-9.8	-52.96	4.35	40th
9 - 1FTD	-10.1	-57.35	2.86	13th
10 - 1LEX	-10.7	-59.82	3.01	17h
11 - 1M6F	-10	-56.55	3.04	18th
12 - 1PRP	-8.2	-47.43	5.9	48th
13 - 1QV4	-12.1	-41.33	2.95	15th
14 - 1QV8	-12.1	-33.59	3.25	19th
15 - 1VZK	-10.2	-52.47	3.89	33rd
16 - 227D	-8.8	-51.58	5.73	47th
17 - 289D	-9.6	-50.46	4.31	39th
18 - 298D	-9.4	-46.15	4.57	42nd
19 - 2DBE	-8	-55.02	6.69	53rd
20 - 302D	-11.2	-38.54	3.85	30th
21 - 311D	-10.8	-52.47	3.89	10th
22 - 328D	-10.5	0[1]	10[1]	57th
23 - 360D	-9.7	-49.77	4.80	44th
24 - 442D	-12	-39.81	2.71	8th
25 - 443D	-12.2	-35.86	2.91	14th
26 - 447D	-11.9	-31.74	3.44	21st
27 - 448D	-11.9	-43.28	2.83	12th
28 - 453D	-12	-27.33	4.09	37th
29 - 102D	-8.2	-50.80	6.40	51st
30 - 121D	-10.4	-60.23	2.57	7th
31 - 195D	-10.2	-68.45	2.53	6th
32 - 1D43	-11.9	-36.64	3.64	26th
33 - 1D44	-11.8	-32.41	3.62	25th
34 - 1D45	-11.7	-31.71	3.93	34th
35 -1D46	-11.9	-33.60	3.52	23rd
36 - 1D63	-8	-48.05	6.19	50th
37 - 1D86	-10.3	-63.59	2.80	11th
38 - 1DNE	-9.9	-54.92	2.77	9th
39 - 1DNH	-11.7	-38.15	3.70	27th
40 - 1DSA	-9.4	-5.82	8.44	54th
41 - 1EEL	-10.2	-45.08	4.03	36th
42 - 1LEY	-10.1	-62.33	2.47	4th
43 - 1ZPH	-11.5	-60.37	2.02	2nd
44 - 1ZPI	-12.1	-59.50	2.29	3rd
45 - 261D	-10.6	-52.65	2.52	5th
46 - 264D	-11.8	-33.99	3.79	28th
47 - 296D	-11.9	-35.18	3.52	22nd
48 - 2B0K	-9.2	-44.48	5.67	46th
49 - 2B3E	-12	-22.02	10[1]	55th
50 - 2DND	-10.3	-46.16	4.38	41st
51 - 2GYX	-11.2	-62.43	1.83	1st
52 - 2I2I	-10.5	-48.85	3.81	29th
53 - 2I5A	-10.3	-51.89	3.88	32nd
54 - 2NLM	-10.3	-51.18	3.98	35th
55 - 303D	-11.2	-37.47	4.59	43rd
56 - 449D	-12.2	-37.59	2.97	16th
57 - 8BNA	-11.9	-34.89	3.58	24th

[1] Docking error (It was not enabled to anchor the ligand in the receptor).

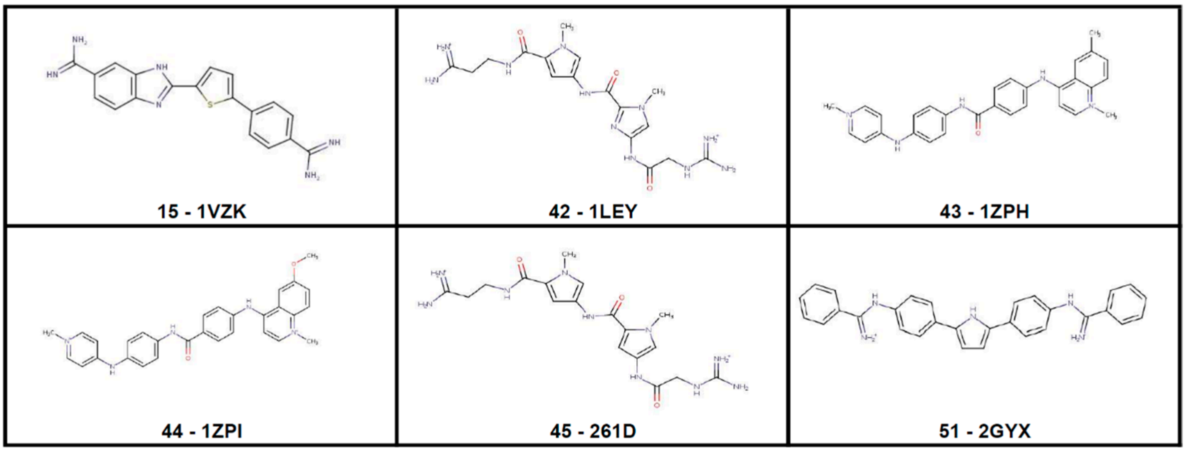

Figure S2. Ligands chosen for MD simulations after docking simulation.

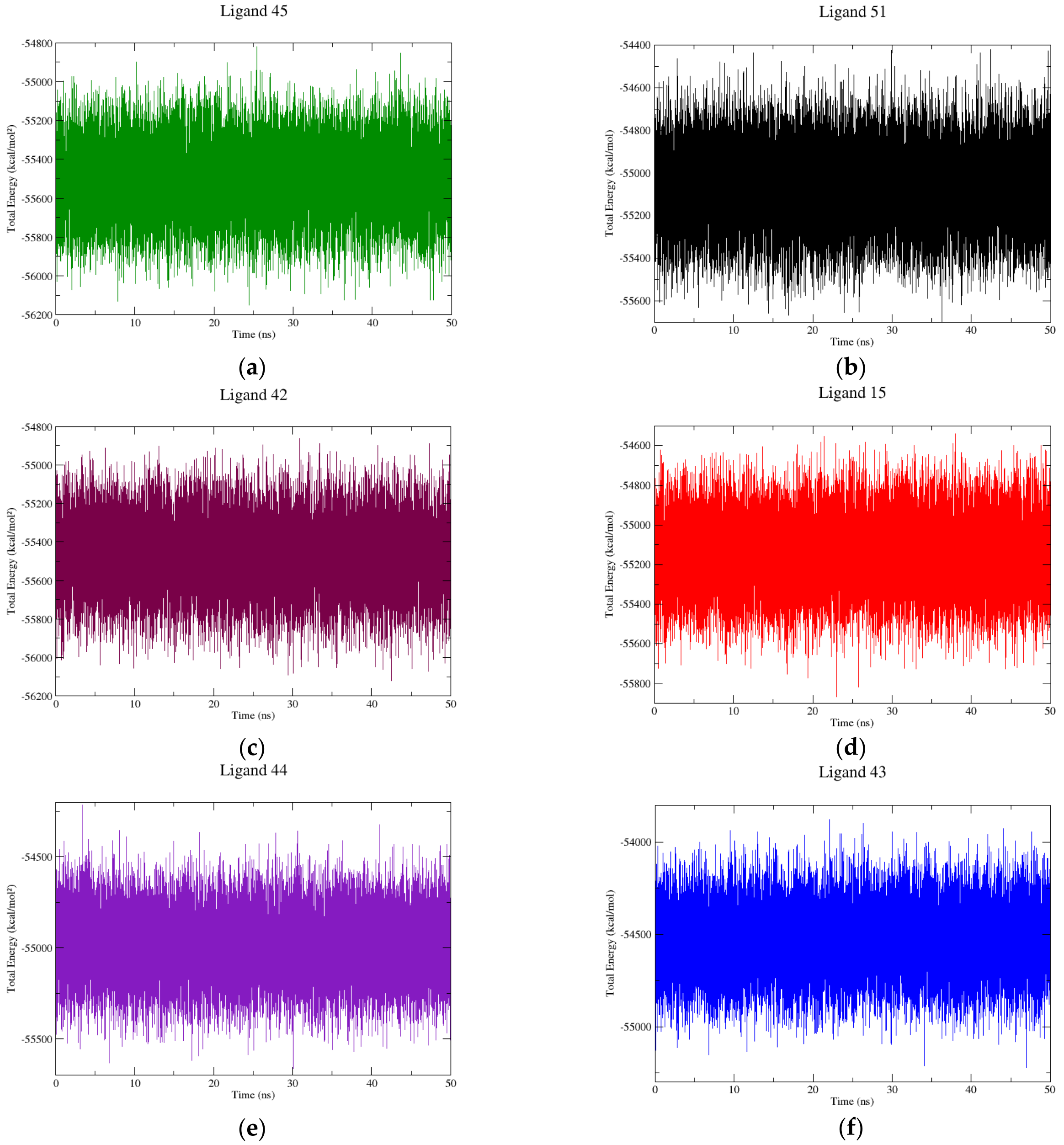

Figure S3. Total Energy (Kcal/mol) calculated in Dynamic Molecular Simulator for interactions with DNA and ligands: (**a**) 45; (**b**) 51; (**c**) 42; (**d**) 15; (**e**) 44; (**f**) 43.
